# Mykotoxine – Bestimmung von Deoxynivalenol und Deepoxydeoxynivalenol in Urin mittels LC-MS/MS: Biomonitoring-Methode

**DOI:** 10.34865/bi5148110d10_2or

**Published:** 2025-06-30

**Authors:** Marion Berger, Lennart Marske, Bernhard Monien, Solveigh Siodlaczek, Thomas Göen, Andrea Hartwig, MAK Commission

**Affiliations:** 1 Bundesanstalt für Arbeitsschutz und Arbeitsmedizin (BAuA). Fachbereich 4 – Gefahrstoffe und biologische Arbeitsstoffe. Gruppe 4.2 – Medizinischer Arbeitsschutz. Biomonitoring Nöldnerstraße 40/42 10317 Berlin Deutschland; 2 Bundesinstitut für Risikobewertung. Fachgruppe 54 – Abt. Lebensmittelsicherheit Max-Dohrn-Straße 8–10 10589 Berlin Deutschland; 3 Friedrich-Alexander-Universität Erlangen-Nürnberg. Institut und Poliklinik für Arbeits-, Sozial- und Umweltmedizin Henkestraße 9–11 91054 Erlangen Deutschland; 4 Institut für Angewandte Biowissenschaften. Abteilung Lebensmittelchemie und Toxikologie. Karlsruher Institut für Technologie (KIT) Adenauerring 20a, Geb. 50.41 76131 Karlsruhe Deutschland; 5 Ständige Senatskommission zur Prüfung gesundheitsschädlicher Arbeitsstoffe. Deutsche Forschungsgemeinschaft, Kennedyallee 40, 53175 Bonn, Deutschland. Weitere Informationen: Ständige Senatskommission zur Prüfung gesundheitsschädlicher Arbeitsstoffe | DFG

**Keywords:** Mykotoxine, Deoxynivalenol, Biomonitoring, Urin, LC-MS/MS, mycotoxins, deoxynivalenol, biomonitoring, urine, LC-MS/MS

## Abstract

The working group “Analyses in Biological Materials” of the German Senate Commission for the Investigation of Health Hazards of Chemical Compounds in the Work Area (MAK Commission) developed and verified the presented biomonitoring method. The aim of this method is the selective and sensitive quantitation of deoxynivalenol (DON; free DON plus glucuronides not otherwise specified) and its metabolite deepoxydeoxynivalenol (DOM‑1) in urine. After enzymatic hydrolysis of the urine sample and purification of the analytes on an immunoaffinity column, followed by preconcentration of the eluates under a stream of nitrogen, determination is carried out by high-performance liquid chromatography-tandem mass spectrometry (LC‑MS/MS). Calibration is performed with comparative standards prepared in urine and treated analogously to the samples to be analysed. DON is quantified using an internal standard (ISTD; ^13^C_15_‑DON), whereas DOM‑1 is quantified without the use of an ISTD. Good precision data with standard deviations below 9% for DON and below 6% for DOM‑1, as well as good accuracy data with mean relative recoveries in the range of 93–114% for DON and 97–103% for DOM‑1, show that the method provides reliable and accurate analytical results. The method is both selective and sensitive, and has a limit of quantitation of 0.179 μg/l for DON and of 0.26 μg/l for DOM‑1. Due to rapid renal excretion, the method is primarily suitable for analysing acute exposure which occurred only hours prior to sampling.

## 1 Kenndaten der Methode

**Table:** 

**Matrix**	Urin		
**Analytisches Messprinzip**	Flüssigkeitschromatographie mit Tandem‑Massenspektrometrie (LC‑MS/MS)		
**Parameter und entsprechender Arbeitsstoff**			
**Arbeitsstoff**	**CAS-Nr.**	**Parameter**	**CAS-Nr.**
Deoxynivalenol (DON)	51481-10-8	Deoxynivalenol (DON)	51481-10-8
		Deepoxydeoxynivalenol (DOM‑1)	88054-24-4

### Zuverlässigkeitskriterien

#### Deoxynivalenol (DON)

**Table:** 

Präzision in der Serie:	Standardabweichung (rel.)	*s_w_* = 4,5 %, 2,9 %, 1,5 %, 1,4 % bzw. 1,1 %
	Streubereich	*u* = 11,5 %, 7,5 %, 3,9 %, 3,7 % bzw. 2,9 %
	bei einer dotierten Konzentration von 0,78 μg, 2,4 μg, 5,6 μg, 13,8 μg oder 16,8 μg DON pro Liter Urin und n = 6 Bestimmungen	
Präzision von Tag zu Tag:	Standardabweichung (rel.)	*s_w_* = 8,5 %, 7,4 %, 3,9 %, 7,9 % bzw. 2,1 %
	Streubereich	*u* = 20,1 %, 17,4 %, 10,0 %, 19,2 % bzw. 5,0 %
	bei einer dotierten Konzentration von 0,78 μg, 2,4 μg, 5,6 μg, 13,8 μg oder 16,8 μg DON pro Liter Urin und n = 6–8 Bestimmungen	
Richtigkeit in der Serie:	Wiederfindung (rel.)	*r* = 103 %, 111 %, 98,8 %, 114 % bzw. 102 %
	bei einer dotierten Konzentration von 0,78 μg, 2,4 μg, 5,6 μg, 13,8 μg oder 16,8 μg DON pro Liter Urin und n = 6 Bestimmungen	
Richtigkeit von Tag zu Tag:	Wiederfindung (rel.)	*r* = 93,3 %, 102 %, 94,9 %, 104 % bzw. 99,5 %
	bei einer dotierten Konzentration von 0,78 μg, 2,4 μg, 5,6 μg, 13,8 μg oder 16,8 μg DON pro Liter Urin und n = 6–8 Bestimmungen	
Nachweisgrenze:	0,049 μg DON pro Liter Urin	
Bestimmungsgrenze:	0,179 μg DON pro Liter Urin	

#### Deepoxydeoxynivalenol (DOM‑1)

**Table:** 

Präzision in der Serie:	Standardabweichung (rel.)	*s_w_* = 3,8 %, 5,0 % bzw. 4,6 %
	Streubereich	*u* = 9,8 %, 12,9 % bzw. 11,7 %
	bei einer dotierten Konzentration von 0,78 μg, 2,4 μg oder 5,6 μg DOM‑1 pro Liter Urin und n = 6 Bestimmungen	
Präzision von Tag zu Tag:	Standardabweichung (rel.)	*s_w_* = 3,2 %, 5,1 % bzw. 5,6 %
	Streubereich	*u* = 7,9 %, 12,5 % bzw. 14,3 %
	bei einer dotierten Konzentration von 0,78 μg, 2,4 μg oder 5,6 μg DOM‑1 pro Liter Urin und n = 6–7 Bestimmungen	
Richtigkeit in der Serie:	Wiederfindung (rel.)	*r* = 102 %, 101 % bzw. 96,9 %
	bei einer dotierten Konzentration von 0,78 μg, 2,4 μg oder 5,6 μg DOM‑1 pro Liter Urin und n = 6 Bestimmungen	
Richtigkeit von Tag zu Tag:	Wiederfindung (rel.)	*r* = 103 %, 103 % bzw. 96,6 %
	bei einer dotierten Konzentration von 0,78 μg, 2,4 μg oder 5,6 μg DOM‑1 pro Liter Urin und n = 6–7 Bestimmungen	
Nachweisgrenze:	0,07 μg DOM‑1 pro Liter Urin	
Bestimmungsgrenze:	0,26 μg DOM‑1 pro Liter Urin	

## 2 Allgemeine Informationen zu Deoxynivalenol

Mykotoxine sind natürliche Substanzen, die als sekundäre Stoffwechselprodukte von Pilzen gebildet werden. Sie kommen häufig in pilzbefallenen Nahrungspflanzen vor (Eskola et al. 2020) und bilden eine chemisch und toxikologisch heterogene Gruppe von Substanzen (Sabbioni et al. 2022), zu der unter anderem Aflatoxine, Ochratoxin A, Gliotoxin, Citrinin (siehe auch die Methode der Kommission: Berger et al. 2025) und Deoxynivalenol (DON) gehören.

DON, auch bekannt als Vomitoxin, ist ein Mykotoxin aus der Klasse der Trichothecene und wird von Pilzen, u. a. der Gattung *Fusarium* gebildet, die Süßgräser wie Weizen, Hafer, Gerste und Mais befallen (SCF 2002). Durch Bindung an Ribosomen inhibiert DON die Proteinbiosynthese. Akute Wirkungen nach Aufnahme von DON sind beim Menschen Erbrechen, Diarrhoe und Schwindel. In Tierversuchen an Mäusen und Schweinen wurden nach chronischer Exposition Wachstumsverzögerungen und eine Dysregulation des Immunsystems beobachtet (Pestka 2010).

Der Mensch resorbiert DON nach oraler Aufnahme vollständig über den Gastrointestinaltrakt und scheidet es innerhalb von 24 h vollständig renal, hauptsächlich in Form der Glucuronide DON‑15‑Glucuronid (DON‑15‑GlcA) und DON‑3‑Glucuronid (DON‑3‑GlcA), wieder aus (Mengelers et al. 2019; Warth et al. 2013). Etwa 66–95 % des über den Urin ausgeschiedenen DON liegt in glucuronidierter Form vor, wobei DON‑15‑GlcA den Hauptmetaboliten darstellt (Vidal et al. 2018). Des Weiteren werden freies DON und der Metabolit Deepoxydeoxynivalenol (DOM‑1) mit dem Urin ausgeschieden (Ali et al. 2016; Deng et al. 2018; Rodríguez-Carrasco et al. 2014). Abbildung 1 zeigt die Strukturformeln von DON und DOM‑1.

**Abb.**
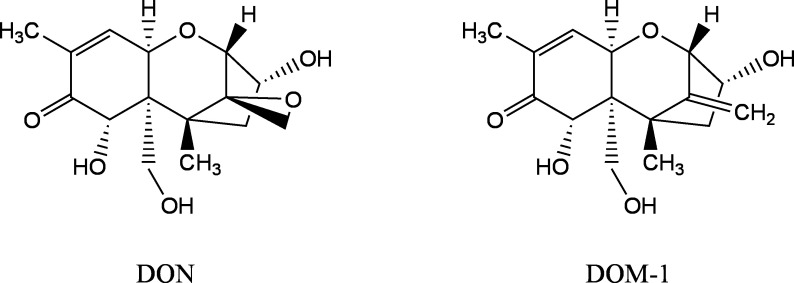
Strukturformeln von DON und dessen Metaboliten DOM‑1

Expositionen der Allgemeinbevölkerung durch den Verzehr von mit DON verunreinigten Lebensmitteln werden häufig beobachtet (Heyndrickx et al. 2015). Die Exposition der europäischen Bevölkerung gegen DON und seine Deri­vate (3-Acetyl-DON, 15-Acetyl-DON und DON-3-Glucosid) wurde aufgrund des weitverbreiteten Vorkommens und der Bedenken hinsichtlich möglicher nega­tiver Auswirkungen auf die menschliche Gesundheit unter anderem im Rahmen des HBM4EU-Projekts analysiert (Keyte et al. 2022; Namorado et al. 2024). Von der Europäischen Behörde für Lebensmittelsicherheit (*European Food Safety Authority*, EFSA) wurde für DON und seine Derivate eine tolerierbare tägliche Aufnahmemenge (*tolerable daily intake*, TDI) von 1 μg/kg Körpergewicht festgesetzt (SCF 2002). In Tabelle 1 sind DON- und DOM‑1-Hinter­grundgehalte im Urin der Allgemeinbevölkerung angegeben.

**Tab.1:** DON- und DOM‑1-Konzentrationen im Urin der Allgemeinbevölkerung

Land/Region (Anzahl der Erwachsenen)	Probe	DON			DOM‑1			Literatur
		Nachweis­häufigkeit [%]^a)^	GM/Median [μg/l]	Bereich [μg/l]	Nachweis­häufigkeit [%]^a)^	Median [μg/l]	Bereich [μg/l]	
Belgien (239)	1. Morgenurin	37	1,7^c)^	< 0,5–130	**–**	**–**	**–**	Heyndrickx et al. 2015
Deutschland (120)	24-h-Urin	98,3	2,66	0,69–17,05^b)^	**–**	**–**	**–**	Namorado et al. 2024
Deutschland (360)	24-h-Urin	99	4,19	< 0,3–99,1	**–**	**–**	**–**	Schmied et al. 2023
Deutschland (50)	1. Morgenurin	100	7,35c)	1,06–38,4	**–**	**–**	**–**	Ali et al. 2016
Europa (1270)	Verschiedene Urinproben	96,1	4,79	0,39–26,1b)	**–**	**–**	**–**	Namorado et al. 2024
Portugal (94)	24-h-Urin	63	2,51c)	< 1,0–36,3	39	0,24	< 0,5–5,13	Martins et al. 2019
Spanien (20)	1. Morgenurin	100	75,6c)	53,0–118,0d)	**–**	**–**	**–**	Gallardo-Ramos et al. 2024

GM: geometrischer Mittelwert

a) Prozentsatz der Messwerte oberhalb der Bestimmungsgrenze

b) 5.–90. Perzentil

c) Median

d) 25.–75. Perzentil

An Arbeitsplätzen erfolgt die Exposition u. a. durch Inhalation belasteter Stäube und wird in der landwirtschaftlichen Produktion und der Lebensmittelproduktion beschrieben (Föllmann et al. 2016; Turner et al. 2010; Viegas et al. 2018). In Tabelle 2 sind beispielhafte Konzentrationen von DON und DOM‑1 im Urin von beruflich Exponierten angegeben.

**Tab.2:** DON- und DOM‑1-Konzentrationen im Urin von beruflich Exponierten

Exponierte, Land (Anzahl, Geschlecht)	Probe	DON			DOM‑1			Literatur
		Nachweis­häufigkeit [%]^a)^	MW ± SD [μg/l]	Bereich [μg/l]	Nachweis­häufigkeit [%]^a)^	MW ± SD [μg/l]	Bereich [μg/l]	
Mühlenarbeiter, Deutschland (12, ♂)	Spontanurin	100	6,50 ± 3,33	3,28–13,8	54	0,105 ± 0,58^b)^	NWG (0,100)–0,216	Föllmann et al. 2016
Mühlenarbeiter, Deutschland (5, ♀)	Spontanurin	100	8,08 ± 4,48	0,850–10,4	60	0,110 ± 0,073^b)^	NWG (0,100)–0,228	
Kontrollen, Deutschland (13, ♂)	Spontanurin	100	6,85 ± 4,47	1,01–14,6	38	0,085 ± 0,049^b)^	NWG (0,100)–0,184	
Arbeiter Getreidesilos, Frankreich (18)	1. Morgenurin	97	16,5^c)^	**–**	**–**	**–**	**–**	Ndaw et al. 2021
	Spontanurin (vor der Schicht)	98	9,90^c)^	**–**	**–**	**–**	**–**	
	Spontanurin (nach der Schicht)	100	22,1^c)^	**–**	**–**	**–**	**–**	
Landwirte, Frankreich (76, ♂)	1. Morgenurin	> 99	6,8^c)^	0,8−28,8	34	0,2^c)^	0,2−2,8	Turner et al. 2010
Arbeiter in einer Brotteigfabrik, Portugal (9 ♀, 12 ♂)	Spontanurin	43^d)^	34,9 ± 17,5^d)^^, ^^e)^	12,6−64,5^d)^^, ^^e)^	**–**	**–**	**–**	Viegas et al. 2018
Kontrollen, Portugal (6 ♀, 12 ♂)	Spontanurin	0	−	< NWG (1,24)−BG (4,14)^d)^^, ^^e)^	–	–	–	

BG: Bestimmungsgrenze; MW: Mittelwert; NWG: Nachweisgrenze; SD: Standardabweichung

a) Prozentsatz der Messwerte oberhalb der BG

b) Werte < NWG wurden als NWG/2 in die Berechnung des Mittelwerts einbezogen.

c) Median

d) DON-Glucuronid

e) μg/g Kreatinin

## 3 Grundlage des Verfahrens

Die hier beschriebene Analysenmethode ermöglicht die Quantifizierung des Mykotoxins DON (freies DON plus nicht weiter spezifizierte Glucuronide) und dessen Metaboliten DOM‑1 in Urin. Die Quantifizierung erfolgt für DON mit inter­nem Standard (ISTD; ^13^C_15_‑DON) und für DOM‑1 ohne ISTD. Nach enzymatischer Hydrolyse der Urinprobe werden die Analyten auf einer Immunoaffinitätssäule angereichet und mit Methanol eluiert. Es folgt eine Aufkonzentrierung der Eluate im Stickstoffstrom und anschließend die Bestimmung der Analyten mittels Hochleistungsflüssigkeit­s­chromatographie-Tandemmassenspektrometrie (LC‑MS/MS). Die Kalibrierung erfolgt mit Vergleichsstandards, die in Urin angesetzt und in der gleichen Weise behandelt werden wie die zu analysierenden Proben.

## 4 Geräte, Chemikalien und Lösungen

### 4.1 Geräte

- HPLC‑Anlage mit binärer Pumpe, Autosampler, Säulenofen und Degasser (z. B. Nexera XR, Shimadzu Deutschland GmbH, Duisburg)
- Triple-Quadrupol-Massenspektrometer (z. B. AB SCIEX QTRAP 5500 mit Elektrosprayionisierung, AB SCIEX Germany GmbH, Darmstadt)
- Analytische HPLC‑Trennsäule (z. B. Kinetex^®^ Core-Shell Technologie; Kinetex^®^ 2,6 μm Biphenyl 100 Å, 100 × 2,1 mm, Phenomenex Ltd. Deutschland, Aschaffenburg)
- UHPLC‑Vorsäule (z. B. Nr. AJO-9209, SecurityGuard ULTRA Cartridges, Biphenyl 2,1 mm ID, inklusive Säulenhalter, Phenomenex Ltd. Deutschland, Aschaffenburg)
- Stickstoffgenerator (z. B. cmc Instruments GmbH, Eschborn)
- Reinstwasseranlage (z. B. Veolia Water Solutions & Technologies, Saint-Maurice, Frankreich)
- Laborzentrifuge (z. B. Fisher Scientific GmbH, Schwerte)
- Analysenwaage (z. B. Sartorius AG, Göttingen)
- pH‑Meter (z. B. Mettler-Toledo GmbH, Gießen)
- Abblasstation (z. B. Biotage Sweden AB, Uppsala, Schweden)
- Ultraschallbad zum Entgasen der Eluenten (z. B. SONOREX SUPER RK 510 H, BANDELIN electronic GmbH & Co. KG, Berlin)
- Rotationsmischer (z. B. Cole-Parmer^TM^ Stuart^TM^, Fisher Scientific GmbH, Schwerte)
- Vortex-Schüttler (z. B. IKA‑Werke GmbH & Co. KG, Staufen)
- Vorrichtung für die Festphasenextraktion (z. B. VisiPrep^TM^ SPE‑Vakuumverteiler, Supelco^®^, Merck KGaA, Darmstadt)
- Inkubator mit Orbital-Shaker (z. B. Cole-Parmer^TM^ Stuart^TM^, Fisher Scientific GmbH, Schwerte)
- Variabel einstellbare Pipetten mit passenden Pipettenspitzen (z. B. Eppendorf AG, Hamburg)
- 2500‑ml‑Glasflaschen mit Schraubverschluss (z. B. DURAN^®^, Schott AG, Mainz)
- Verschiedene Messkolben und Bechergläser (z. B. DURAN^®^, Schott AG, Mainz)
- 0,2‑μm‑Spritzenvorsatzfilter (13 mm, regenerierte Zellulose) (z. B. CS – Chromatographie Service GmbH, Langerwehe)
- 15‑ml‑Polypropylen‑Zentrifugenröhrchen mit konischem Boden, graduiert (z. B. COTECH Vertriebs GmbH, Berlin)
- 13‑ml‑Polypropylen‑Zentrifugenröhrchen mit rundem Boden (z. B. COTECH Vertriebs GmbH, Berlin)
- 5‑ml‑Luer-Lock-Einmalspritzen mit Einmal-Injektionskanülen (z. B. Omnifix^®^ Luer Solo, B. Braun SE, Melsungen)
- 1,5‑ml‑Polypropylen‑Gewindefläschchen mit Schraubkappen (z. B. MACHEREY-NAGEL GmbH & Co. KG, Düren)
- 2‑ml‑Polypropylen‑Reaktionsgefäße (z. B. Eppendorf AG, Hamburg)
- Urinbecher aus Polypropylen (z. B. Sarstedt AG & Co. KG, Nümbrecht)

### 4.2 Chemikalien

Wenn nicht anders angegeben, sind alle genannten Chemikalien mindestens in p. a.‑Qualität zu verwenden.

#### Referenzstandards und ISTD

- Deoxynivalenol (DON), 100 mg/l in Acetonitril (z. B. Nr.  34124, Supelco^®^, Merck KGaA, Darmstadt)
- Deepoxydeoxynivalenol (DOM‑1), 50 mg/l in Acetonitril (z. B. Nr. 10003662 (S02033), Romer Labs Division Holding GmbH, Getzersdorf, Österreich)
- ^13^C_15_‑Deoxynivalenol, 25 mg/l in Acetonitril (z. B. Nr. DRE-A12147100AL-25, LGC Standards GmbH, Wesel)

#### Sonstige Chemikalien

- Immunoaffinitätssäule IAC DONStarR, Lagerung bei 4 °C (z. B. Nr. 10001970, Romer Labs Division Holding GmbH, Getzersdorf, Österreich)
- Ammoniumacetat (z. B. Nr. 15681570, Honeywell Fluka^TM^, Fisher Scientific GmbH, Schwerte)
- Dinatriumhydrogenphosphat-Dihydrat (z. B. Nr. 137036, Merck KGaA, Darmstadt)
- *Escherichia coliβ*‑Glucuronidase, K12, ≥ 140 U/mg bei 37 °C (z. B. Nr. 03708446103, Roche Diagnostics Deutschland GmbH, Mannheim)
- Essigsäure LiChropur^®^, 100 % (z. B. Nr. 533001, Supelco^®^, Merck KGaA, Darmstadt)
- Isopropanol LiChrosolv^®^, u. a. für die Hinterkolbenspülung der Pumpen (z. B. Nr. 102781, Supelco^®^, Merck KGaA, Darmstadt)
- Kaliumdihydrogenphosphat (z. B. Nr. 137039, Merck KGaA, Darmstadt)
- Methanol LiChrosolv^®^, ≥ 99,97 % (z. B. Nr. 106035, Supelco^®^, Merck KGaA, Darmstadt)
- PBS‑Tabletten Calbiochem^®^ (z. B. Nr. 524650, Merck KGaA, Darmstadt)
- Hochreines Wasser (z. B. Veolia Water Solutions & Technologies, Saint-Maurice, Frankreich)
- Nativer Urin von Freiwilligen mit möglichst geringen DON‑ und DOM‑1‑Hintergrundgehalten

### 4.3 Lösungen

- Stammlösung A für Phosphatpuffer nach Sørensen (pH 6,8)
  In einen 1000‑ml‑Messkolben werden 9,078 g Kaliumdihydrogenphosphat eingewogen und in etwas hochreinem Wasser gelöst. Anschließend wird der Kolben bis zur Markierung mit hochreinem Wasser aufgefüllt und die Lösung gut gemischt.
- Stammlösung B für Phosphatpuffer nach Sørensen (pH 6,8)
  In einen 1000‑ml‑Messkolben werden 11,876 g Dinatriumhydrogenphosphat-Dihydrat eingewogen und in etwas hochreinem Wasser gelöst. Anschließend wird der Kolben bis zur Markierung mit hochreinem Wasser aufgefüllt und die Lösung gut gemischt.
- Phosphatpuffer nach Sørensen (pH 6,8)
  In einen 100‑ml‑Messkolben werden 49,2 ml der Stammlösung B pipettiert. Anschließend wird der Kolben mit Stammlösung A auf 100 ml aufgefüllt und die Lösung gut gemischt.

Der Phosphatpuffer nach Sørensen ist bei 4 °C mindestens eine Woche stabil.

- Eluent A
  In einen 1000‑ml‑Messkolben werden 77,08 mg Ammoniumacetat eingewogen und in etwas hochreinem Wasser gelöst. Anschließend wird 1 ml Essigsäure in den Kolben pipettiert und dieser mit hochreinem Wasser bis zur Markierung aufgefüllt.
- Eluent B
  In einen 1000‑ml‑Messkolben werden 77,08 mg Ammoniumacetat eingewogen und in etwas Methanol gelöst. Anschließend wird 1 ml Essigsäure in den Kolben pipettiert und dieser mit Methanol bis zur Markierung aufgefüllt.
- Gradientenlösung (Eluent A ∶ Eluent B; 98 ∶ 2 (V ∶ V))
  In einen 100‑ml‑Messkolben werden 2 ml Eluent B vorgelegt, anschließend wird der Messkolben mit Eluent A bis zur Markierung aufgefüllt.
- PBS‑Puffer (pH 7,4)
  Eine PBS‑Tablette wird in ein 800‑ml‑Becherglas gegeben und mit ca. 500 ml hochreinem Wasser versetzt. Das Becherglas wird anschließend in ein Ultraschallbad gestellt, bis sich die Tablette vollständig aufgelöst hat. Anschließend wird die Lösung in einen 1000‑ml‑Messkolben überführt, wobei das Becherglas mehrmals mit hochreinem Wasser nachgespült wird. Zuletzt wird der Messkolben mit hochreinem Wasser bis zur Markierung aufgefüllt und die Lösung gut gemischt.

Der PBS‑Puffer ist bei Raumtemperatur mindestens sechs Monate stabil.

### 4.4 ISTD

- ^13^C_15_‑DON‑Dotierlösung (150 μg/l)
  In einem 2‑ml‑Polypropylen‑Reaktionsgefäß werden 6 μl der Referenzstandardlösung ^13^C_15_‑DON (25 μg/ml in Acetonitril) mit 994 μl hochreinem Wasser versetzt, anschließend wird die Lösung gut gemischt.

Die ^13^C_15_‑DON‑Dotierlösung ist vier Tage bei −20 °C stabil.

### 4.5 Kalibrierstandards

- Kalibrierstandard‑Dotierlösung (150 μg Analyt/l)
  In ein 2‑ml‑Polypropylen‑Reaktionsgefäß werden 1,5 μl der DON‑Standardlösung (100 mg/l) und 3 μl der DOM‑1‑Standardlösung (50 mg/l) pipettiert und mit 995 μl hochreinem Wasser versetzt, anschließend wird die Lösung gut gemischt.

Die Kalibrierstandard‑Dotierlösung ist bei −20 °C vier Tage stabil.

Die Kalibrierstandards werden gemäß dem in Tabelle 3 angegebenen Pipettierschema angesetzt. Die Kalibrier­standard‑Dotierlösung wird jeweils zu 2,5 ml möglichst unbelastetem nativem Urin in ein 13‑ml‑Polypropylen‑Zentrifugenröhrchen mit rundem Boden gegeben. Der Urin zum Ansetzen der Kalibrierstandards sollte möglichst geringe Hintergrundgehalte an DON und DOM‑1 aufweisen. Die Entwickler der Methode haben hierfür den Urin einer freiwilligen Person verwendet, die fünf Tage auf den Verzehr von Getreideprodukten verzichtet hatte.

**Tab.3:** Pipettierschema zur Herstellung der Kalibrierstandards für die Bestimmung von DON und DOM‑1 in Urin

Lösung	Kalibrierstandard‑Dotierlösung [μl]	Analytkonzentration [μg/l]	^13^C_15_‑DON‑Dotierlösung [μl]	^13^C_15_‑DON-Konzentration [μg/l]
DB^a)^	0	0	0	0
B^b)^	0	0	25	1,5
K1	5	0,3	25	1,5
K2	10	0,6	25	1,5
K3	15	0,9	25	1,5
K4	20	1,2	25	1,5
K5	25	1,5	25	1,5
K6	35	2,1	25	1,5
K7	50	3	25	1,5
K8	100	6	25	1,5
K9	150	9	25	1,5
K10	200	12	25	1,5
K11	250	15	25	1,5
K12	300	18	25	1,5
K13	350	21	25	1,5

a) Doppelblindprobe

b) Blindprobe

### 4.6 Kontrollstandardlösung

Zur Überprüfung des equilibrierten Messsystems wird die Messung einer Kontrollstandardlösung eingesetzt (Über­prüfung von Druck, Peakintensität und Retentionszeit). Alle Proben einer Serie werden am Anfang und am Ende der Serie von Kontrollstandardlösungen eingeschlossen.

- Kontrollstandardlösung (7 μg DON/DOM‑1/^13^C_15_‑DON/l)
  In einem 1,5‑ml‑Polypropylen‑Gewindefläschchen mit Schraubkappe werden 46,7 μl der Kalibrierstandard‑­Dotierlösung (150 μg/l) und 46,7 μl der ^13^C_15_‑DON‑Dotierlösung (150 μg/l) mit 907 μl der Gradienten­lösung versetzt und die Lösung anschließend gut gemischt.

Die Kontrollstandardlösung wird bei −20 °C gelagert und wöchentlich frisch angesetzt.

## 5 Probenahme, Probenaufbereitung und Aufreinigung mit der Immunoaffinitätssäule

### 5.1 Probenahme

Die Urinproben werden in Urinbechern aus Polypropylen gesammelt, aliquotiert und bis zur Probenaufbereitung bei −20 °C gelagert.

### 5.2 Probenaufbereitung

Vor der Probenaufbereitung werden die Urinproben auf Raumtemperatur gebracht und homogenisiert. 2,5 ml der Urinproben werden in 13‑ml‑Rundboden‑Zentrifugenröhrchen gegeben, mit 25 μl der ^13^C_15_‑DON‑Dotierlösung versetzt und gut durchmischt. Anschließend werden die Proben für die enzymatische Hydrolyse mit 2,5 ml Phosphatpuffer (pH 6,8) und 40 μl *β*‑Glucuronidase versetzt und für 20 Stunden bei 37 °C und 210 U/min in einem Inkubator geschüttelt. Nach der Hydrolyse werden die Proben bei 2045 × *g* und 10 °C für 15 min zentrifugiert. Die Überstände werden jeweils in ein neues 13‑ml‑Rundboden‑Zentrifugenröhrchen dekantiert.

### 5.3 Aufreinigung mit der Immunoaffinitätssäule

Die Anreicherung von DON und DOM‑1 erfolgt mit einer Immunoaffinitätssäule in Verbindung mit einem SPE‑Vakuum­kammersystem. Die stationäre Phase der Immunoaffinitätssäule ist ein Gel, das mit analytspezifischen Antikörpern gekoppelt ist. Eine Konditionierung der Säule vor der Aufgabe der Urinprobe ist nicht erforderlich. Nachdem Säule und Urinprobe auf Raumtemperatur gebracht wurden, wird die Probe zur Anreicherung und Aufreinigung portionsweise auf die Säule gegeben. Die Probenaufgabe erfolgt ohne Anlegen eines Vakuums. Die Flussrate beträgt ca. 1 ml/min. Anschließend wird die Säule mit 2 × 2,5 ml PBS‑Puffer gewaschen. In der Säule verbliebene Flüssigkeitsreste werden nach dem Waschen unter leichtem Druck von oben entfernt, wobei die Säule nicht austrocknen darf. Für die Elution der gebundenen Analyten wird wasserfreies Methanol verwendet. In ein graduiertes 15‑ml‑Polypropylen‑Zentrifugenröhrchen mit konischem Boden, in dem 200 μl hochreines Wasser vorgelegt sind, wird mit 2 × 1,5 ml Methanol eluiert. Die ersten 1,5 ml Methanol werden vor dem Eluieren einige Sekunden auf der Säule belassen. In der Säule verbliebene Methanolreste werden durch Anlegen eines leichten Überdruckes eluiert. Bei 40 °C wird die Probe unter Stickstoffstrom auf 200 μl eingeengt, mit 300 μl Gradientenlösung versetzt, auf einem Vortex-Schüttler homogenisiert und über einen 0,2‑μm‑Spritzenfilter in ein 1,5‑ml‑Polypropylen‑Gewindefläschchen mit Schraubkappe filtriert.

## 6 Instrumentelle Arbeitsbedingungen

Die analytischen Messungen erfolgten an einer Gerätekonfiguration bestehend aus einem Flüssigkeitschromato­graphen (Nexera XR, Shimadzu Deutschland GmbH, Duisburg) und einem Tandem-Massenspektrometer (AB SCIEX QTRAP 5500, AB SCIEX Germany GmbH, Darmstadt).

### 6.1 Hochleistungsflüssigkeitschromatographie

**Table:** 

HPLC Säule:	Kinetex^®^ Biphenyl; 2.6 μm; 100 × 2.1 mm
Vorsäule:	UHPLC Vorsäule Biphenyl 2.1 mm ID
Temperatur des Säulenofens:	40 °C
Temperatur des Autosamplers	15 °C
Injektionsvolumen:	20 μl
Eluent A:	0.1% Essigsäure und 1 mM Ammoniumacetat in hochreinem Wasser
Eluent B:	0.1% Essigsäure und 1 mM Ammoniumacetat in Methanol
Gradientenprogramm:	siehe Tabelle 4

**Tab.4:** Gradientenprogramm für die Bestimmung von DON und DOM‑1 in Urin

Zeit [min]	Flussrate [ml/min]	Eluent A [%]	Eluent B [%]
0,01	0,45	98	2
2	0,45	98	2
5	0,45	20	80
5,2	0,45	2	98
8	0,45	2	98
8,01	0,45	98	2
11	0,45	98	2

### 6.2 Tandem-Massenspektrometrie

**Table:** 

Quelle:	TurboSpray
Ionisierungsmodus:	ESI, negativ
Ionenspray‑Spannung:	−4500 V
Quellentemperatur:	500 °C
Nebuliser‑Gas:	Stickstoff, 80 psi (551.58 kPa)
Turbo‑Heater‑Gas:	Stickstoff, 80 psi (551.58 kPa)
Curtain‑Gas:	Stickstoff, 35 psi (241.32 kPa)
Kollisionsgas:	Stickstoff
Scan‑Modus:	*Multiple Reaction Monitoring* (MRM)
Dwell‑Time:	70 msec
Parameterspezifische Einstellungen:	siehe Tabelle 5

Die Retentionszeiten in Tabelle 5 können nur als Anhaltspunkt dienen. Der Anwender hat sich selbst von der Trennleistung der verwendeten Säule und dem daraus resultierenden Retentionsverhalten der Analyten zu überzeugen.

**Tab.5:** MRM‑Parameter und Retentionszeiten für die Bestimmung von DON und DOM‑1 in Urin

Analyt/ ISTD	Retentionszeit [min]	Vorläufer‑Ion (*m/z*)	Produkt‑Ion (*m/z*)	Declustering-Potenzial [V]	Eintritts-Potenzial [V]	Kollisionsenergie [V]	Zellaustritts-Potenzial [V]
DON	4,81	355,2	59^a)^	−45	−10	−52	−5
			295,1	−45	−10	−14	−19
			265,1	−45	−10	−20	−17
DOM‑1	5,12	339,2	59^a)^	−70	−10	−20	−9
			249,2	−70	−10	−16	−17
^13^C_15_‑DON	4,81	370,3	59,1^a)^	−40	−10	−40	−7
			310,3	−40	−10	−14	−29

a) Quantifier

## 7 Analytische Bestimmung

Zur analytischen Bestimmung von DON und DOM‑1 in Urin werden jeweils 20 µl der nach Abschnitt 5 aufgearbeiteten Probe in das HPLC‑MS/MS-System injiziert und unter den in Abschnitt 6 angegebenen Bedingungen analysiert. Die analytische Trennung erfolgt nach Durchlaufen einer UHPLC‑Biphenyl‑Vorsäule auf einer Kinetex^®^ Biphenyl‑Säule. Die Identifizierung der Analyten DON und DOM‑1 erfolgt anhand der Retentionszeiten und spezifischen Massenübergänge (siehe Tabelle 5).

**Abb.2:**
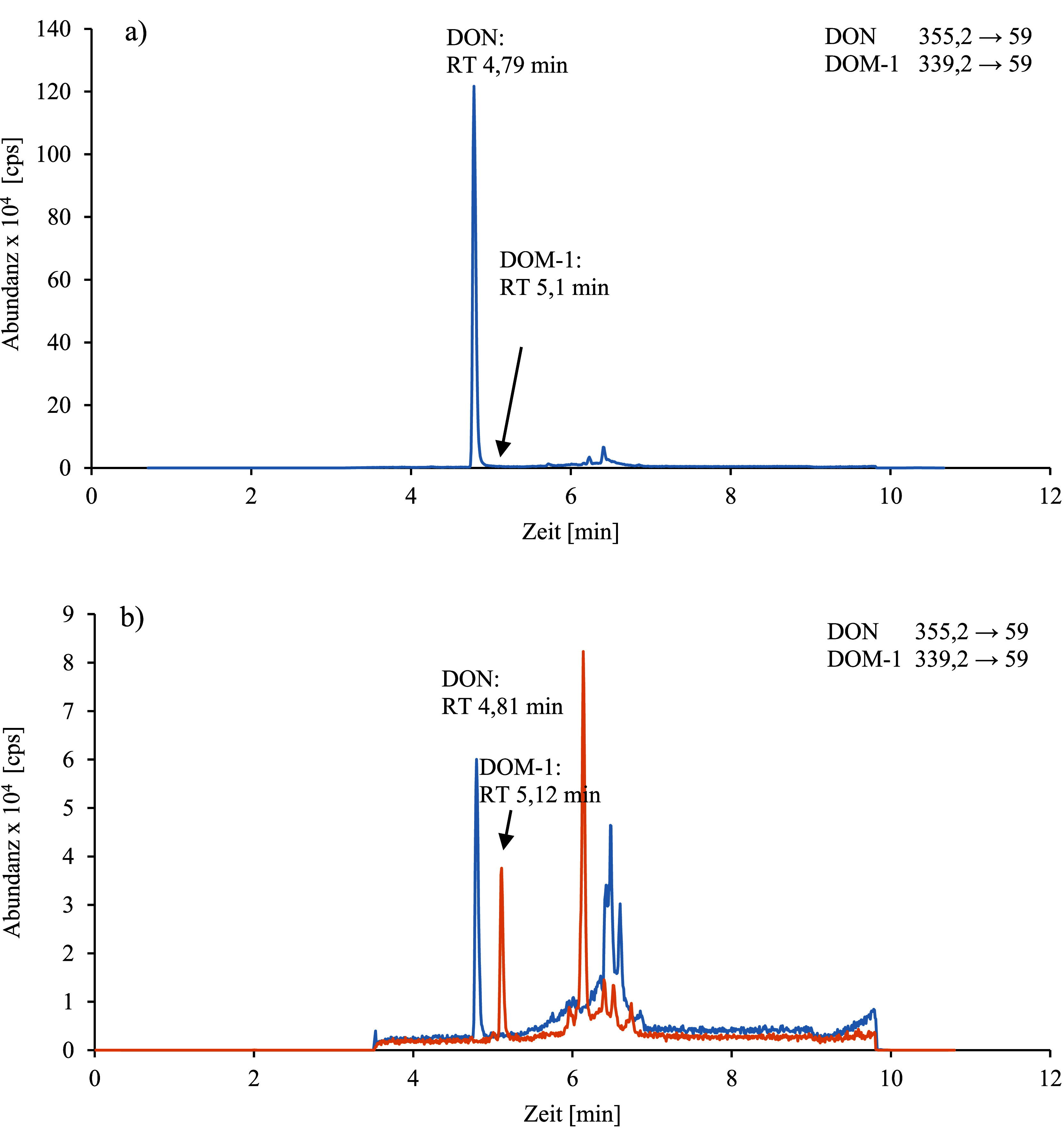
Chromatogramm a) einer nativen Urinprobe mit 14,4 μg DON/l und einer DOM‑1‑Konzentration unterhalb der Bestimmungsgrenze und b) eines Kalibrierstandards mit 0,3 μg DON sowie 0,3 μg DOM‑1 pro Liter Urin

Abbildung 2 zeigt repräsentative Chromatogramme a) einer nativen Urinprobe mit einer ermittelten Konzentration für DON von 14,4 μg/l Urin sowie einer Konzentration von DOM‑1 unterhalb der Bestimmungsgrenze und b) eines Kalibrierstandards, der mit DON und DOM‑1 in einer Konzentration von jeweils 0,3 μg/l Urin dotiert ist.

## 8 Kalibrierung

Zur Kalibrierung der Methode werden die gemäß Abschnitt 4.5 hergestellten Kalibrierlösungen analog zu den Urin­proben aufgearbeitet (vgl. Abschnitt 5), allerdings ohne weitere Zugabe von ISTD, und mittels HPLC‑MS/MS (vgl. Abschnitt 6) analysiert. Die Kalibriergerade für DON wird durch das Auftragen des Peakflächenverhältnisses von DON und ^13^C_15_‑DON gegen das Konzentrationsverhältnis von DON und ^13^C_15_‑DON erstellt. Die Kalibriergerade für DOM‑1 wird durch das Auftragen der Peakfläche gegen die dotierte DOM‑1‑Konzentration erstellt.

**Abb.3:**
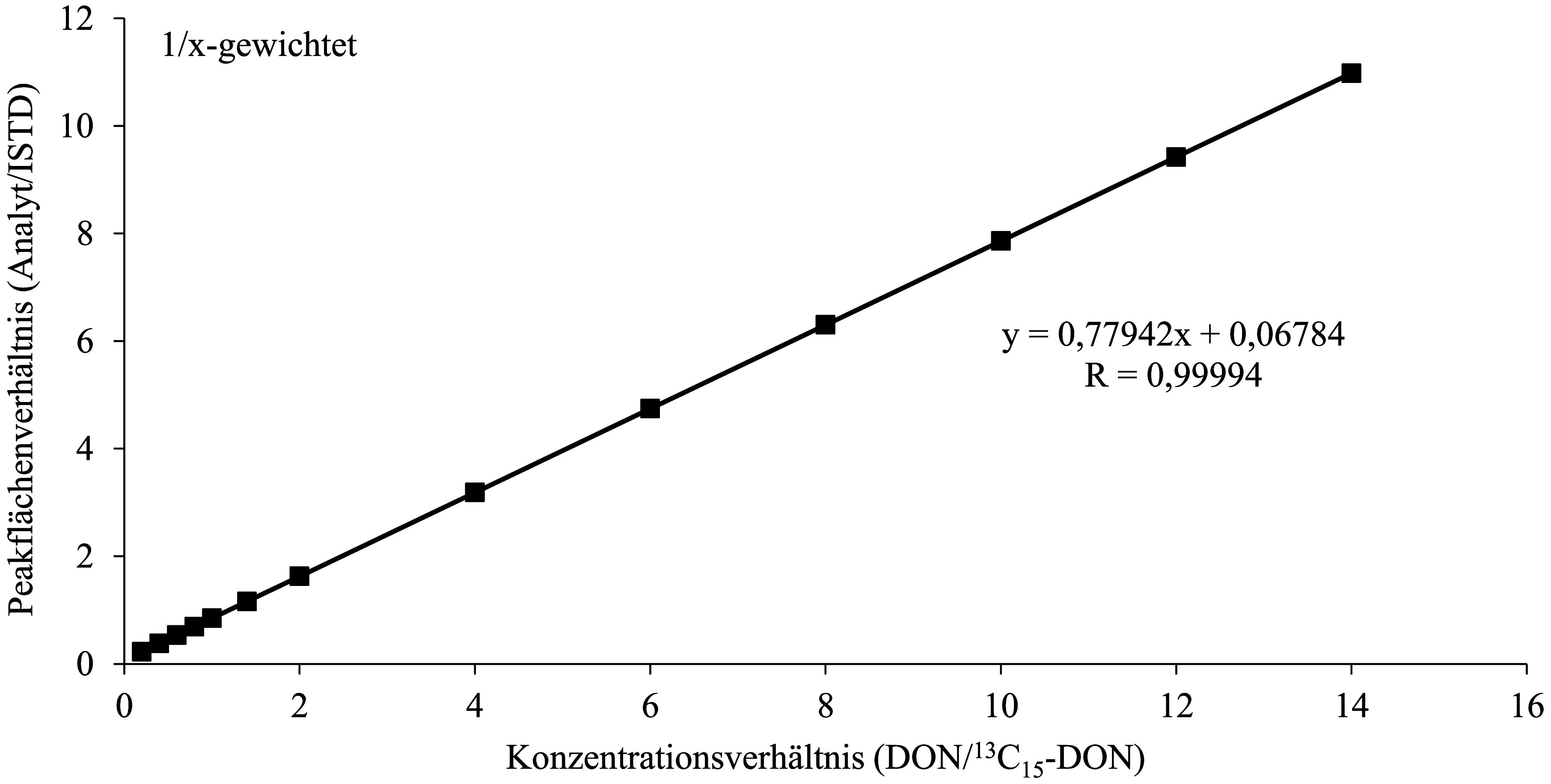
Kalibriergerade für die Bestimmung von DON in Urin

Die jeweiligen Kalibrierbereiche sind in Tabelle 6 dargestellt. Die Daten wurden mit einer linearen Funktion unter 1/x‑Wichtung angepasst. Für beide Analyten ergaben sich in den untersuchten Konzentrationsbereichen Korrelations­koeffizienten von R ≥ 0,999. Abbildung 3 und 4 zeigen beispielhaft Kalibriergeraden für die Bestimmung von DON und DOM‑1 in Urin.

**Tab.6:** Kalibrierbereiche für die Bestimmung von DON und DOM‑1 in Urin

Analyt	Kalibrierbereich [μg/l]	ISTD	ISTD [μg/l]
DON	0,3–21	^13^C_15_‑DON	1,5
DOM‑1	0,3–6	–	–

**Abb.4:**
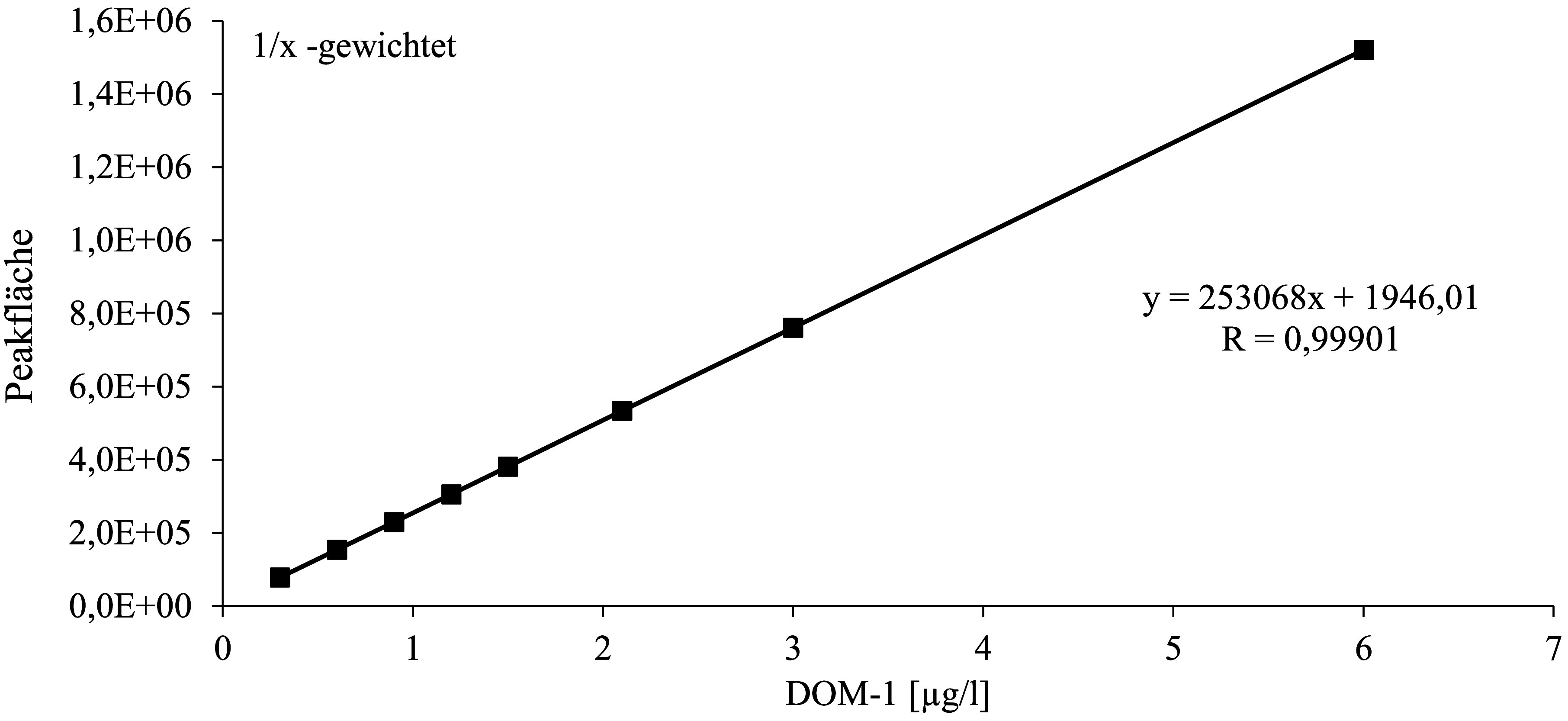
Kalibriergerade für die Bestimmung von DOM‑1 in Urin

## 9 Berechnung der Analysenergebnisse

Zur Berechnung der DON-Konzentration einer Urinprobe wird der Quotient aus der Peakfläche des Analyten und der Peakfläche des ISTDs ^13^C_15_‑DON gebildet. Mithilfe der zur Analysenserie gehörigen Kalibrierfunktion (vgl. Abschnitt 8) kann aus dem ermittelten Quotienten der Analytgehalt in μg/l Urin berechnet werden.

Die DOM‑1-Konzentration einer Urinprobe wird aus der ermittelten Peakfläche mit Hilfe der zur Analysenserie gehö­rigen Kalibrierfunktion (vgl. Abschnitt 8) in μg/l Urin berechnet.

Der Kalibrierbereich muss gegebenenfalls an die zu erwartenden Konzentrationen angepasst werden.

## 10 Standardisierung der Messergebnisse und Qualitätssicherung

Zur Sicherung der Qualität der Analysenergebnisse wird gemäß den Richtlinien der Bundesärztekammer und den Angaben in dem von der Kommission veröffentlichten allgemeinen Kapitel verfahren (Bader et al. 2010; Bundes­ärztekammer 2014).

Zur Qualitätssicherung werden mit jeder Kalibrierreihe Blind- und Doppelblindproben in Urin angesetzt (siehe Tabelle 3). Die Blindproben werden mit ISTD dotiert, aber nicht mit den Analyten. Die Doppelblindproben enthalten weder Analyt noch ISTD. Zudem wird in jeder Analysenserie ein Reagenzienleerwert (hochreines Wasser anstelle der Urinprobe) aufgearbeitet und gemessen.

Zur Präzisionskontrolle werden in jeder Analysenserie mindestens zwei Qualitätskontrollproben mituntersucht, die eine bekannte Konzentration der Analyten aufweisen. Da käufliches Material nicht zur Verfügung steht, muss dieses Kontrollmaterial selbst hergestellt werden, indem Urin mit den Analyten im relevanten Konzentrationsbereich dotiert wird (siehe Tabelle 7). Zusätzlich werden in jeder Analysenserie die Kontrollstandardlösung (siehe Abschnitt 4.6) sowie Gradientenlösung (siehe Abschnitt 4.3) gemessen und es erfolgen Spülschritte mit Methanol.

**Tab.7:** Pipettierschema zur Herstellung der Qualitätskontrollproben für die Bestimmung von DON und DOM‑1 in Urin

Qualitätskontrollproben	Urin [μl]	Kalibrierstandard-Dotierlösung [μl]	Analytkonzentration [μg/l]	^13^C_15_‑DON-Dotierlösung [μl]	^13^C_15_‑DON-Konzentration [μg/l]
QC_0,78_	ad 2500	13	0,78	25	1,5
QC_2,4_		40	2,4	25	
QC_5,6_		93	5,6	25	
QC_13,8_		230	13,8	25	
QC_16,8_		280	16,8	25	

## 11 Beurteilung des Verfahrens

Die Zuverlässigkeit des Verfahrens wurde durch eine umfassende Validierung sowie durch Nachstellung und Prüfung der Methode in einem zweiten, unabhängigen Labor bestätigt. Zur Ermittlung der Präzision und Richtigkeit der Methode wurden die Qualitätskontrollproben (siehe Tabelle 7) verwendet, die, wie in Abschnitt 5 und 6 beschrieben, aufgearbeitet und analysiert wurden.

Zur Ermittlung der Präzision und Richtigkeit der Methode wurden für DOM‑1 nur die Qualitätskontrollproben QC_0,78_, QC_2,4 _und QC_5,6 _einbezogen.

### 11.1 Präzision

Zur Bestimmung der Präzision in der Serie wurden die hergestellten Qualitätskontrollmaterialien je Konzentration sechsfach parallel aufgearbeitet und analysiert. Die hieraus ermittelten Präzisionsdaten sind in Tabelle 8 dargestellt.

**Tab.8:** Präzision in der Serie für die Bestimmung von DON und DOM‑1 in Urin

Analyt	Dotierte Konzentration [μg/l]	Anzahl n	Gemessene Konzentration [μg/l]	Standardabweichung (rel.) * s_w_* [%]	Streubereich *u* [%]
DON	0,78	6	0,81	4,5	11,5
	2,4	6	2,6	2,9	7,5
	5,6	6	5,5	1,5	3,9
	13,8	6	15,7	1,4	3,7
	16,8	6	17,1	1,1	2,9
DOM‑1	0,78	6	0,8	3,8	9,8
	2,4	6	2,4	5,0	12,9
	5,6	6	5,4	4,6	11,7

Zur Bestimmung der Präzision von Tag zu Tag wurden die hergestellten Qualitätskontrollmaterialien für DON an sechs bis acht verschiedenen Tagen aufgearbeitet und analysiert. Für DOM‑1 erfolgte die Aufarbeitung und Analyse der Qualitätskontrollproben an sechs bis sieben verschiedenen Tagen. Die so ermittelten Präzisionsdaten sind in Tabelle 9 dargestellt.

**Tab.9:** Präzision von Tag zu Tag für die Bestimmung von DON und DOM‑1 in Urin

Analyt	Dotierte Konzentration [μg/l]	Anzahl n	Gemessene Konzentration [μg/l]	Standardabweichung (rel.) *s_w_* [%]	Streubereich *u* [%]
DON	0,78	8	0,73	8,5	20,1
	2,4	8	2,5	7,4	17,4
	5,6	6	5,3	3,9	10,0
	13,8	7	14,3	7,9	19,2
	16,8	7	16,7	2,1	5,0
DOM‑1	0,78	7	0,81	3,2	7,9
	2,4	7	2,5	5,1	12,5
	5,6	6	5,4	5,6	14,3

### 11.2 Richtigkeit

Die Richtigkeit des Verfahrens wurde aus den Daten zur Bestimmung der Präzision in der Serie und der Präzision von Tag zu Tag (vgl. Abschnitt 11.1) ermittelt. Die jeweils errechneten mittleren relativen Wiederfindungen für DON und DOM‑1 sind in Tabelle 10 aufgeführt.

**Tab.10:** Mittlere relative Wiederfindung für die Bestimmung von DON und DOM‑1 in Urin

Analyt	Dotierte Konzentration [μg/l]	Präzision in der Serie			Präzision von Tag zu Tag		
		Anzahl n	Wiederfindung (rel.) *r* [%]	Bereich [%]	Anzahl n	Wiederfindung (rel.) *r* [%]	Bereich [%]
DON	0,78	6	103	98,0–110	8	93,3	85,7–105
	2,4	6	111	106–114	8	102	93,5–114
	5,6	6	98,8	96,2–100	6	94,9	89,1–98,8
	13,8	6	114	112–115	7	104	89,7–114
	16,8	6	102	100–103	7	99,5	95,6–102
DOM‑1	0,78	6	102	98,6–108	7	103	99,0–110
	2,4	6	101	92,1–108	7	103	94,7–110
	5,6	6	96,9	89,4–102	6	96,6	91,6–104

### 11.3 Nachweis- und Bestimmungsgrenzen

Die in Tabelle 11 aufgeführten Nachweis- und Bestimmungsgrenzen wurden mit der Kalibriergeradenmethode nach DIN 32645 ermittelt. Dies erfolgte bei DON auf Grundlage der sechs untersten Kalibrierpunkte und bei DOM‑1 auf Grundlage aller acht Kalibrierpunkte (DIN 2008). Aufgrund der Komplexität der Urinmatrix, welche erheblichen Schwankungen unterliegen kann, wurden die Bestimmungsgrenzen angehoben.

**Tab.11:** Nachweis- und Bestimmungsgrenzen für die Bestimmung von DON und DOM‑1 in Urin

Analyt	Nachweisgrenze [μg/l]	Bestimmungsgrenze [μg/l]
DON	0,049	0,179
DOM‑1	0,070	0,260

### 11.4 Analytstabilität in der Urinmatrix

Die Analytstabilität in der Urinmatrix wurde bei Raumtemperatur, bei 4 °C und bei −20 °C untersucht. Die Stabilität bei Raumtemperatur wurde für DON und DOM‑1 über einen Zeitraum von 24 Stunden untersucht und ist für die Probenvorbereitung relevant. Die Stabilität während der Lagerung im Kühlschrank bei 4 °C wurde über einen Zeitraum von 48 Stunden untersucht und ist für eine Kurzzeitlagerung von Urinproben relevant. Die Stabilität bei −20 °C ist für eine längere Probenlagerung wichtig und wurde nach einer Woche, nach zwei, nach fünf und nach 13 Wochen bestimmt.

Zur Bestimmung der Analytstabilität wurden die Qualitätskontrollproben einfach aufgearbeitet und analysiert. Als Akzeptanzkriterium wurde die Entscheidung 2002/657/EG der Europäischen Union zugrunde gelegt, welche eine Abweichung vom Nominalwert von −50 bis +20 % erlaubt (Europäische Kommission 2002). Die ermittelten relativen Wiederfindungen von DON und DOM‑1 in Urin nach Lagerung bei Raumtemperatur und 4 °C lagen im Akzeptanzbereich (Tabelle 12).

**Tab.12:** Analytstabilität von DON und DOM‑1 in Urin bei Raumtemperatur und 4 °C

Analyt	Dotierte Konzentration [μg/l]	Relative Wiederfindung [%] nach Lagerung bei	
		Raumtemperatur für 24 h	4 °C für 48 h
DON	0,78	86,7	87,5
	2,4	99,2	92,3
	13,8	99,9	96,4
	16,8	103	97,5
DOM‑1	0,78	89,1	113
	2,4	91,1	110

Die relative Wiederfindung der Analyten nach Lagerung bei −20 °C ist in Tabelle 13 dargestellt. Abgesehen von der Wiederfindung von DOM‑1 nach einer Woche Lagerung lagen alle Wiederfindungen im Akzeptanzbereich.

**Tab.13:** Analytstabilität von DON und DOM‑1 in Urin bei −20 °C

Analyt	Dotierte Konzentration [μg/l]	Relative Wiederfindung [%] nach Lagerung bei −20 °C für			
		**1 Woche**	2 Wochen	5 Wochen	13 Wochen
DON	0,78	86,5	79,4	77,2	91,1
	2,4	94,1	89,6	86,0	87,7
	13,8	96,8	92,9	89,6	85,9
	16,8	100	93,8	89,8	89,5
DOM‑1	0,78	121	113	109	108
	2,4	130	104	115	91,3

### 11.5 Analytstabilität im Extrakt

Die Analytstabilität im Extrakt der aufgearbeiteten Qualitätskontrollproben wurde nach Lagerung bei −20 °C für eine Woche sowie für zwei, fünf und 13 Wochen untersucht. Als Akzeptanzkriterium wurde die Entscheidung 2002/657/EG der Europäischen Union zugrunde gelegt, welche eine Abweichung vom Nominalwert von −50 bis +20 % erlaubt (Europäische Kommission 2002). Die Ergebnisse zur Wiederfindung im Extrakt nach Lagerung bei −20 °C sind in Tabelle 14 dargestellt. Die Wiederfindung für DOM‑1 war nach der ersten Woche Lagerung und für die Qualitäts­kontrollprobe QC_0,78 _nach zwei Wochen Lagerung nicht im Akzeptanzbereich. Auch die Wiederfindung für DON in der Qualitätskontrollprobe QC_0,78 _nach 13 Wochen Lagerung war nicht im Akzeptanzbereich.

**Tab.14:** Analytstabilität von DON und DOM‑1 im Extrakt bei −20 °C

Analyt	Dotierte Konzentration [μg/l]	Relative Wiederfindung [%] nach Lagerung bei −20 °C für			
		1 Woche	2 Wochen	5 Wochen	13 Wochen
DON	0,78	91,7	88,2	90,0	123
	2,4	99,3	101	103	115
	13,8	102	106	103	113
	16,8	104	103	102	112
DOM‑1	0,78	129	127	112	89,8
	2,4	122	113	106	85,4

### 11.6 Störeinflüsse

Mit dieser Methode wird der DON‑Gesamtgehalt (freies DON plus Glucuronide) nach Hydrolyse der Glucuronide sowie die DOM‑1-Konzentration in Urin analysiert. Aufgrund der raschen renalen Ausscheidung eignet sich die Methode vorwiegend für den Nachweis einer akuten Exposition wenige Stunden vor der Probenahme.

Im Rahmen der Methodenentwicklung wurden verschiedene SPE‑Materialien zur Aufreinigung und Anreicherung der Analyten getestet. Weder mit C18‑Materialien noch mit den in verschiedenen Publikationen beschriebenen Polymer­phasen konnten zufriedenstellende Ergebnisse bezüglich Nachweis- und Bestimmungsgrenzen erzielt werden.

Durch den Einsatz einer Immunoaffinitätssäule konnten die in der Literatur beschriebenen Nachweis- und Bestim­mungsgrenzen erreicht werden. Die Auswertung von DOM‑1 erfolgte ohne ISTD, weil bei Ver­wendung eines isotopenmarkierten Standards im untersuchten Konzentrationsbereich der geforderte Korrelations­koeffizient von R ≥ 0,995 für die Kalibriergerade nicht erreicht wurde.

Für die Herstellung der Kalibrierstandards wurden Urine verschiedener Personen auf die Gehalte an den zu messenden Analyten getestet. Da in fast allen untersuchten Urinproben nicht vernachlässigbare Gehalte an DON zu finden waren, konnte kein Poolurin verwendet werden. Die Kalibrierstandards wurden schließlich in einem nahezu unbelasteten nativen Urin angesetzt, der von einer Person stammte, die sich vor der Probenahme fünf Tage getreidefrei ernährt hatte. Dieser Urin wurde gesammelt und bei −20 °C gelagert.

## 12 Diskussion der Methode

Die Methode ermöglicht die zuverlässige Bestimmung von DON und dessen Metaboliten DOM‑1 in Urin. Die Vali­dierungsdaten zeigen eine gute Empfindlichkeit, Reproduzierbarkeit und Richtigkeit der Methode, die aus dem Einsatz einer effizienten Probenaufbereitung unter Verwendung einer Immunoaffinitätssäule resultieren. Die stationäre Phase, ein Gel aus Dextranen oder quervernetzter Agarose, ist mit einem geeigneten Liganden (Antikörper) gekoppelt, der die Analyten spezifisch bindet. Die in der eingesetzten Immunoaffinitätssäule verwendeten Antikörper zeigten eine hohe Spezifizität für DON. Die chromatographischen Ergebnisse waren hervorragend; es ergaben sich keine störenden Begleitsignale und es wurden sehr hohe Wiederfindungen erreicht. Die von den Entwicklern der Methode beschriebene Analyse von DOM‑1 konnte von den Prüfern der Methode nicht nachgestellt werden, da die verwendete Immunoaffinitätssäule (IAC DONStarR, Romer Labs Division Holding GmbH, Österreich) im April 2021 vom Hersteller nicht mehr vertrieben und durch eine andere Säule (SH-DonStar IAC, Spezifität 2500 ng DON, Nr. 10001974, Romer Labs Division Holding GmbH, Österreich) ersetzt wurde, die nur für DON spezifisch ist. Die Methode weist eine hohe Sensitivität auf und ist für DON in einem weiten Bereich (bis 21 μg/l Urin) linear, sodass die Methode sowohl für die Anwendung im umweltmedizinischen als auch im arbeitsmedizinischen Bereich geeignet ist.

Zu Beginn der Validierung wurden die Arbeitsbereiche für beide Analyten auf 0,3 bis 3 μg/l festgelegt. Daraus resultierten Konzentrationen für die untersuchten Qualitätskontrollproben für beide Analyten von 0,78 μg/l Urin und 2,4 μg/l Urin und für den ISTD von 1,5 μg/l Urin. Während der Validierung wurden die Arbeitsbereiche erweitert und an Realproben eines laufendenden Forschungsprojektes angepasst (DON: 0,3 bis 21 μg/l Urin, DOM‑1: 0,3 bis 6 μg/l Urin). Daraus resultierten die zusätzlich untersuchten Qualitätskontrollproben für DOM‑1 mit einer Konzentration von 5,6 μg/l Urin und für DON mit 5,6 μg/l, 13,8 μg/l, und 16,8 μg/l Urin. Die Konzentration des ISTD wurde nicht verändert.

**Verwendete Messgeräte** HPLC‑Anlage mit binärer Pumpe, Autosampler, Säulenofen und Degasser (Nexera XR, Shimadzu Deutschland GmbH, Duisburg); Triple-Quadrupol-Massenspektrometer (Modell AB SCIEX QTRAP 5500 mit Elektrosprayionisation, AB SCIEX Germany GmbH, Darmstadt)
